# Piezoelectric Actuator with Frequency Characteristics for a Middle-Ear Implant

**DOI:** 10.3390/s18061694

**Published:** 2018-05-24

**Authors:** Dong Ho Shin, Jin-Ho Cho

**Affiliations:** Institute of Biomedical Engineering Research, Kyungpook National University, 680, Gukchaebosang-ro, Jung-gu, Daegu 41944, Korea; swap9552@naver.com

**Keywords:** piezoelectric actuator, round window (RW), implantable middle-ear hearing aid, finite element analysis

## Abstract

The design and implementation of a novel piezoelectric-based actuator for an implantable middle-ear hearing aid is described in this paper. The proposed actuator has excellent low-frequency output characteristics, and can generate high output in a specific frequency band by adjusting the mechanical resonance. The actuator consists of a piezoelectric element, a miniature bellows, a cantilever membrane, a metal ring support, a ceramic tip, and titanium housing. The optimal structure of the cantilever-membrane design, which determines the frequency characteristics of the piezoelectric actuator, was derived through finite element analysis. Based on the results, the piezoelectric actuator was implemented, and its performance was verified through a cadaveric experiment. It was confirmed that the proposed actuator provides better performance than currently used actuators, in terms of frequency characteristics.

## 1. Introduction

To date, a range of actuators has been developed for implantable middle-ear devices to treat hearing loss [1,2,3]. Middle-ear hearing aids can provide a high level of sound-quality judgment and speech intelligibility, providing a positive experience for hearing-impaired patients, and research is ongoing to improve the performance of actuators in middle-ear hearing aids [4,5]. Typical implantable middle-ear hearing aids include the Vibrant Soundbridge (VSB) by MED-EL Inc. (Innsbruck, Austria), and the Carina device by Cochlear, Ltd (Sydney, Australia). Generally, a middle-ear hearing aid transmits vibrations to the inner ear by contacting the ossicles using an actuator [6,7,8]; however, this method cannot be used in patients with hearing loss who have lost their ossicles due to inherited or acquired diseases. Recently, to overcome this limitation, a method of directly applying vibrational force through the round window (RW) was studied [9].

In the case of the VSB, methods for installing the small actuator, a floating mass transducer (FMT), in the RW niche so as to stimulate the RW membrane were widely studied [10,11,12]. The size of the FMT is sufficiently small that it can enter the RW niche, and as such, it is easy to implant; however, a number of RW-stimulation experiments using the FMT demonstrated poor output characteristics in the low-frequency band (<1 kHz) [13,14]. In the case of the Carina device, the ball tip of the actuator is installed on the RW membrane, and directly stimulates the membrane [15,16]. This actuator demonstrated better output characteristics in the low-frequency band than the FMT in RW-stimulation experiments. However, this actuator is too large to be installed into the middle-ear cavity; instead, it is installed on the outer wall of the temporal bone. Therefore, implantation of this actuator requires a high level of surgical skill [17]. To overcome these limitations in conventional actuators, it is necessary to develop a new actuator for RW stimulation.

In this paper, we described the design of a new actuator based on piezoelectricity. The actuator consists of a piezoelectric element, a miniature bellows, a cantilever membrane, a metal ring support, a ceramic tip, and titanium housing. The proposed actuator is small enough to be easily installed in the RW niche, and has excellent output characteristics in the low-frequency band. Furthermore, to generate high output in a specific frequency band, mechanical resonance was generated by inserting a cantilever membrane inside the actuator. To derive the optimal frequency characteristics of the piezoelectric actuator, finite element analysis (FEA) was performed on the geometric shape of the cantilever membrane; based on the FEA results, the piezoelectric actuator was implemented. Finally, the feasibility of the implemented actuator was verified through a cadaveric experiment.

## 2. Materials and Methods

### 2.1. RW-Drive Middle-Ear Hearing Aid

**Figure 1:**
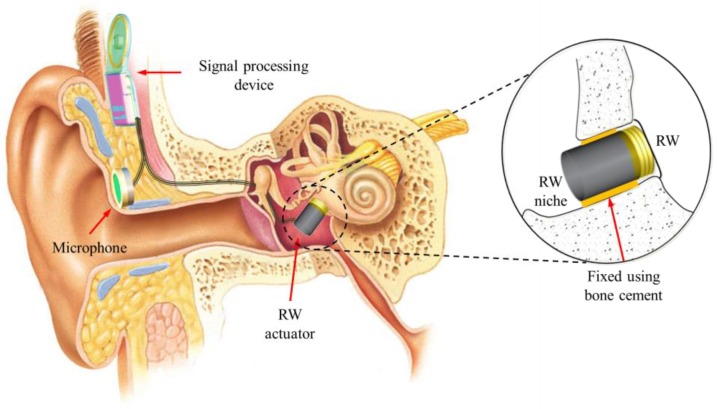
Diagram of the round-window-drive (RW-drive) implantable middle-ear hearing aid [18].

The RW-drive middle-ear hearing aid consists of a microphone, a signal-processing device, and an RW actuator; a conceptual illustration is shown in Figure 1 [18]. The principle of the RW-drive middle-ear hearing aid consists of several steps. Firstly, the audio signal is collected by the microphone, and converted into an electrical signal. Secondly, the converted electrical signal is fit by the signal-processing device based on the patient’s condition, and then transmitted to the actuator. Thirdly, the actuator generates a vibrational signal from the electrical signal, which is transmitted to the cochlea via the RW membrane. Fourthly, this vibrational signal generates a pressure difference between the scala vestibuli and the scala tympani; from this pressure difference, a bioelectrical signal is generated inside the cochlea. Finally, the cerebrum perceives the audio signal from the bioelectrical signal.

### 2.2. Design of the Piezoelectric Actuator

**Figure 2:**
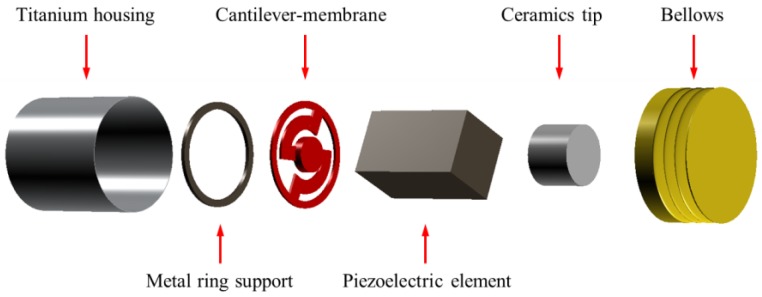
Exploded view of the proposed piezoelectric actuator.

The actuator used in the RW-drive hearing aid must be small due to the spatial limitations of the RW niche. Therefore, based on the anatomical structure of the RW niche [19], the maximum size of the proposed actuator was limited to a diameter of 1.75 mm, and a length of 2.5 mm. An enlarged view of the proposed piezoelectric actuator is shown in Figure 2. The components of the proposed piezoelectric actuator are as follows: titanium housing, a metal ring support for ensuring space between the cantilever membrane and the bottom of the titanium case, a cantilever membrane for generating mechanical resonance, a piezoelectric element, a ceramic tip for connecting the piezoelectric element to the bellows, and a miniature bellows with three corrugations.

Implantable middle-ear hearing aids are used in the treatment of mild–moderate mixed or conductive hearing loss. These devices are also widely used to treat sensorineural hearing loss [20]. The hearing level of patients with sensorineural hearing loss typically drops markedly at frequencies above 1 kHz. Therefore, an implantable middle-ear hearing aid should compensate well for hearing loss in this range. To satisfy this requirement, an actuator used in an implantable middle-ear hearing aid should exhibit mechanical resonance between 2 kHz and 3 kHz. The mechanical resonance of the proposed actuator in this paper was designed at 2.5 kHz.

The piezoelectric actuator for the RW-drive middle-ear hearing aid was designed as described below. When a circular flat membrane is used, the stiffness of the membrane is increased due to the small diameter of the actuator housing. As a result, the displacement generated in the piezoelectric element is reduced. To solve this problem, a bellows with very low stiffness was used [18,21]. Since the stiffness of the bellows was slight when compared with that of the internal cantilever membrane, the frequency characteristics of the actuator were not affected. 

The formulas for calculating stiffness of the bellows and the cantilever membrane are shown in Equations (1) and (2), respectively [18,22].

(1) $$k_{b}=\frac{1.7D_{b}E_{b}t_{b}^{3}n}{w_{b}^{3}C_{b}N},$$

where *k_b_* is the stiffness of the bellows, *D_b_* is the outer diameter of the bellows, *E_b_* is the Young’s modulus describing the elasticity of the material used for the bellows, *t_b_* is the thickness of one ply of the material used for the bellows, *w_b_* is the depth of each corrugation, *C_b_* is a correction factor for the bellows’ stiffness, *n* is the number of layers of the material used for the bellows, and *N* is the number of corrugations in the bellows.

(2) $$k_{c}=\frac{nE_{c}I_{c}W_{c}t_{c}^{3}}{4l_{c}^{3}},$$

where *k_c_* is the stiffness of the cantilever membrane, *n* is the number of arms in the cantilever membrane, *E_c_* is Young’s modulus describing the elasticity of the material used for the cantilever membrane, *I_c_* is the area moment of inertia for the arm of the cantilever membrane, *W_c_* is the width of the arm of the cantilever-membrane, *t_c_* is the thickness of the material used for the cantilever membrane, and *l_c_* is the length of the arm of the cantilever membrane. 

**Figure 3:**
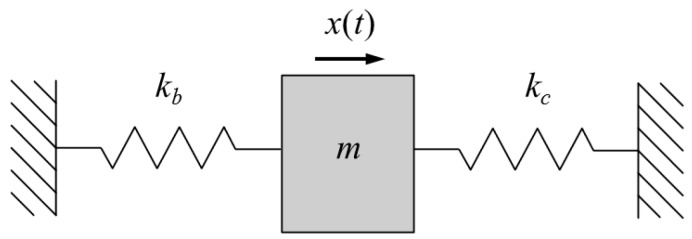
Simple, equivalent mechanical model of the piezoelectric actuator.

The parameters exerting the greatest influence on the stiffness of the bellows and of the cantilever membrane were the thickness of the materials (*t_b_* and *t_c_*), and the deformation area (*w_b_* and *l_c_*) of the materials (where the Young’s moduli of both materials were nearly identical). The values of *t_b_* and *w_b_* were 0.5 mm and 0.0076 mm, respectively, and the values of *t_c_* and *l_c_* were 1 mm and 0.04 mm, respectively. The stiffness of the cantilever membrane was about 70 times greater than that of the bellows, despite only two parameters being compared. The piezoelectric actuator can be represented by a simple, equivalent mechanical model consisting of two springs and a mass (Figure 3).

The theoretically calculated amplitude of the vibration displacement is given by the following Equation:

(3) $$m\ddot{x}\left(t\right)+\left(k_{b}+k_{c}\right)x\left(t\right)=Fcos\omega \left(t\right).$$

Here, *m* is the mass of the piezoelectric element and ceramic tip, *k_b_* is the stiffness of the bellows, *k_c_* is the stiffness of the cantilever membrane, and *F* is the force of the piezoelectric element generated by the current.

The stiffness (*k_actuator_*) and resonant frequency (ω*_r_*) of the piezoelectric actuator are given in Equations (4) and (5), respectively.

(4) $$k_{actuator}=k_{b}+k_{c}$$

(5) $$\omega_{r}=\sqrt{\frac{k_{actuator}}{m}}\;$$

**Figure 4:**
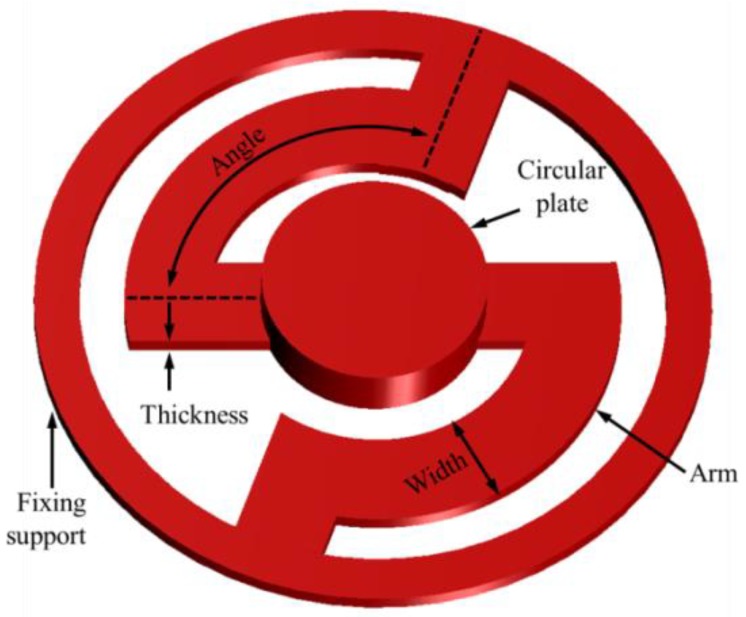
The geometrical shape of the cantilever membrane.

However, the stiffness of the bellows was negligible, because the stiffness of the cantilever was very large when compared with that of the bellows. The piezoelectric element used in the actuator did not exhibit mechanical resonance within the audible frequency range (20 Hz to 20 kHz). Therefore, the frequency characteristics of the piezoelectric actuator were determined by the cantilever membrane. The structure of the cantilever membrane involved a fixing support and a circular plate connected by two arms, as shown in Figure 4. The mechanical resonance of the cantilever membrane could be controlled by the angle, thickness, and width of the two arms [22].

**Figure 5:**
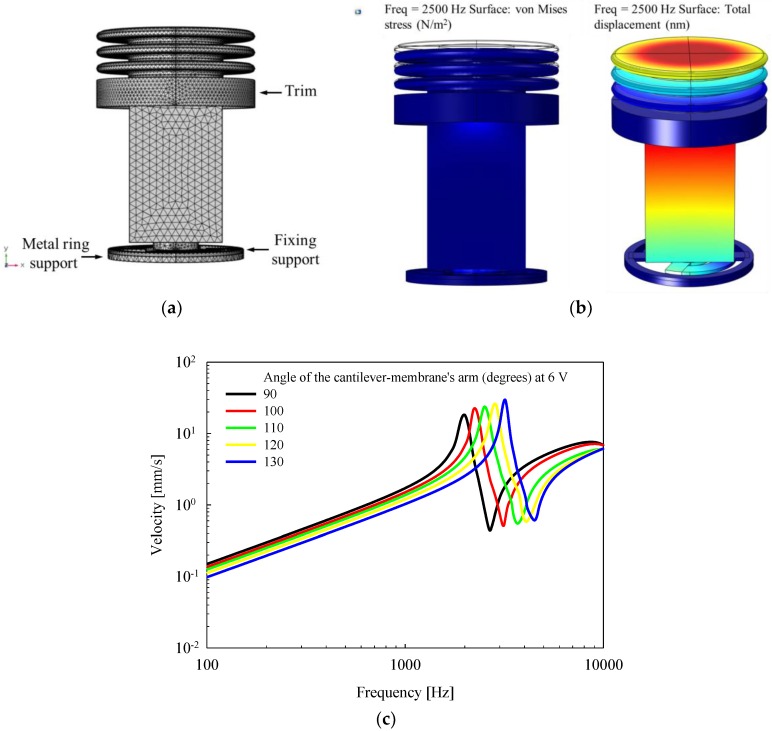
(**a**) Mesh modeling for modal analysis; (**b**) von Mises stress distribution (right), and total displacement distribution (left) of the actuator at 2.5 kHz; (**c**) vibrational characteristics according to the angle of the arm of the cantilever membrane.

To determine the optimal vibrational characteristics of the proposed actuator, vibration analysis of the actuator was performed using the FEA software (COMSOL Multiphysics 5.0; COMSOL Inc., Stockholm, Sweden). Figure 5a shows a three-dimensional model of the piezoelectric actuator used when performing modal analysis. Here, the titanium housing was not represented because, when the actuator is installed in the RW niche of the temporal bone, the housing of the actuator is completely fixed to the wall of the RW niche using bone cement. Therefore, the titanium housing has no effect on the vibrational characteristics of the actuator. All components (except for the titanium housing) of the actuator were established using the solid mechanics routine of the structural mechanics module, and then combined using the “form union” command. The trim, the fixing support, and the metal ring support for coupling with the titanium housing were fixed using a defined “fixed constraint”. The prescribed displacements of the piezoelectric element, the cantilever membrane (except for the fixing support), the bellows, and the ceramic tip were set to “free/free”. The mesh of the actuator model consisted of 116,860 domain elements, 48,864 boundary elements, and 6348 edge elements using a defined “free tetrahedral”. The material properties of the piezoelectric element (lead zirconate titanate, PZT-8) were applied using a defined “material” in the FEA software. The bellows (nickel alloy), the ceramic tip (Al_2_O_3_), the cantilever membrane (stainless steel 316, SUS316), and the metal ring support (SUS316) used in the modal analysis had the following characteristics: density of the bellows, 8900 kg/m^3^; Poisson’s ratio of the bellows, 0.35; Young’s modulus of the bellows, 216.577e^9^ N/m^2^; density of the ceramic tip, 3800 kg/m^3^; Poisson’s ratio of the ceramic tip, 0.22; Young’s modulus of the ceramic tip, 375e^9^ N/m^2^; density of the cantilever membrane and metal ring support, 8070 kg/m^3^; Poisson’s ratio of the cantilever membrane and metal ring support, 0.275; and Young’s modulus of the cantilever membrane and metal ring support, 205e^9^ N/m^2^.

The vibrational frequency characteristics of the piezoelectric actuator were derived using modal analysis, as described below. The piezoelectric element was designed to generate a constant displacement of 300 nm in the vertical direction at 6 V using the piezoelectric effect. To minimize the overall length of the actuator, the thickness and width of the arms of the cantilever membrane were fixed at 0.04 mm and 0.2 mm, respectively. Modal analysis was then performed while increasing the angle of the arm of the cantilever membrane arm from 90° to 130°, in 10° increments. Figure 5b shows the von Mises stress distribution (right), and total displacement distribution (left) of the actuator at 2.5 kHz, obtained from the results of the modal analysis. The vibration magnitude of the piezoelectric actuator, according to the frequency characteristics, was calculated at the center point of the top surface of the bellows. Figure 5c shows the vibrational characteristics of the piezoelectric actuator according to the angle of the arm of the cantilever membrane. From the results of the analysis, mechanical resonance of the actuator occurred at 2.5 kHz when the angle of the arm of the cantilever membrane was 110°.

## 3. Results

### 3.1. Implementation of the Piezoelectric Actuator

**Figure 6:**
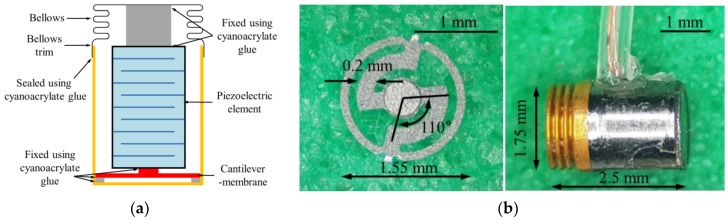
(**a**) Cross section of the piezoelectric actuator; (**b**) cantilever membrane (left) fabricated through etching, and assembled piezoelectric actuator (right).

The piezoelectric actuator was fabricated based on the results of the analysis of the cantilever membrane. The piezoelectric element was a commercial product (PAZ-10-0079) from Murata Manufacturing Co., Ltd. (Kyoto, Japan), with dimensions of 0.9 mm (width) × 0.9 mm (height) × 1.6 mm (depth). The bellows was custom-made by Servometer Inc. (Cedar Grove, NJ, USA), with dimensions of 1.75 mm (diameter) × 0.8 mm (length, including trim length of 0.3 mm). The cantilever membrane (diameter of 1.55 mm, and thickness of 0.04 mm), and metal ring support (diameter of 1.55 mm, and thickness of 0.06 mm) were fabricated by etching stainless steel 316 (SUS316) with chemical solutions. The titanium housing (diameter of 1.75 mm, and length of 2.0 mm, Ti-6Al-4V), and ceramic tip (diameter of 0.6 mm, and length of 0.6 mm, Al_2_O_3_) were fabricated using CNC machining. The components of the actuator were combined using a precision assembly process. All assembly processes were performed in a probe station equipped with a microscope, according to the procedure described below. Firstly, the fixing support of the cantilever membrane, and the metal ring support were combined using cyanoacrylate glue. Secondly, the bottom of the housing, and the metal ring support were secured using cyanoacrylate glue. Thirdly, the bottom of the piezoelectric element, and the circular plate of the cantilever membrane were combined using cyanoacrylate glue. Fourthly, the piezoelectric elements, ceramic tips, and bellows were joined using cyanoacrylate glue. Finally, the trim of the bellows, and the housing were sealed using cyanoacrylate glue (see cross section in Figure 6a). Figure 6b shows a photograph of the cantilever membrane (left) fabricated through etching, and the assembled piezoelectric actuator (right). 

### 3.2. Measurement of Vibrational Characteristics

To confirm the frequency characteristics of the piezoelectric actuator, a vibration measurement experiment was carried out under no-load conditions (i.e., on the bench). All measurements were made using an FFT-based (sampling rates: 96 kHz, number of FFTs: 8192) points data acquisition system (DAQ; NI PXI-4461 board in an NI PXI-1042; National Instruments Co., Austin, TX, USA). The system synchronously measured the vibrational response of the actuator using a laser Doppler vibrometer (LDV; OFV-551 and OFV-5000; Polytec GmbH, Waldbronn, Germany), while generating a sinusoidal signal to drive the piezoelectric actuator. The LDV signal was measured 10 times, on average, at each stimulus frequency.

**Figure 7:**
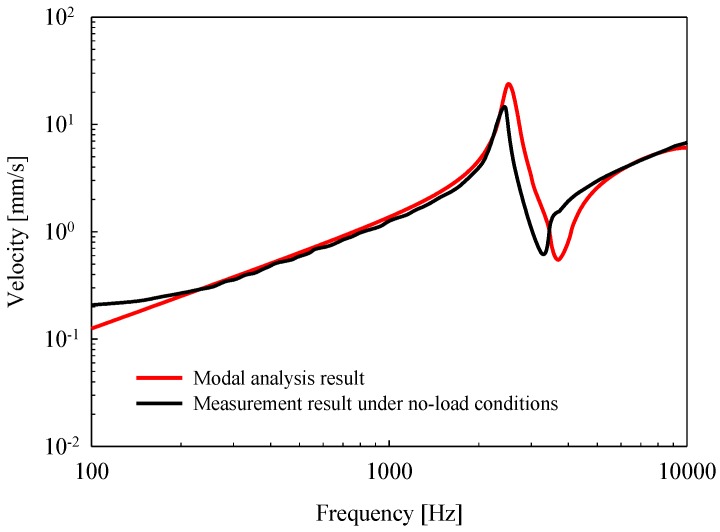
Comparison of vibrational characteristics between the implemented piezoelectric actuator (black solid line), and the results of the modal analysis (red solid line) under no-load conditions.

Vibration measurements were performed on an anti-vibration table, with the bottom of the housing of the piezoelectric actuator fixed firmly to the table using cyanoacrylate glue. Then, after applying a constant voltage of 6 V to the piezoelectric actuator, the frequency characteristics (frequency range: 0.1 kHz to 10 kHz) were measured using the LDV. The vibrational characteristics of the piezoelectric actuator according to frequency are shown in Figure 7. The mechanical resonance of the actuator measured by the LDV was 2.4 kHz (black solid line), and exhibited similar characteristics to those observed in the modal analysis (red solid line). However, the mechanical resonance of the implemented piezoelectric actuator shifted to the low-frequency band, which was considered to be an error generated during the fabrication process.

### 3.3. Cadaveric Experiment

To verify the usefulness of the proposed piezoelectric actuator, a cadaveric experiment was performed using a temporal bone. The frozen temporal bone used was from an 80-year-old male (Caucasian), obtained from the Anatomy Gifts Registry (Hanover, MD, USA). The middle-ear cavity was opened through posterior tympanotomy, with the removal of bony protrusions kept to a minimum, while tissue from the external ear canal was completely removed. The ear canal was replaced by an artificial ear canal (length of 25 mm), made using a syringe, with the tympanic membrane (TM) remaining intact, and one end of the syringe shielded with a cover glass. Sound was injected into the ear canal using an ER-2 insert earphone (Etymotic Research Inc., Elk Grove Village, IL, USA), and the sound pressure of the ear canal was measured 2 mm in front of the TM using a probe microphone (ER 7C; Etymotic Research Inc., Elk Grove Village, IL, USA). To measure the vibrational characteristics of the stapes using the LDV, a reflective bead was attached to the footplate of the stapes. The angle of incidence of the LDV laser did not exceed 60° from the perpendicular to the footplate of the stapes, in accordance with the ASTM-F2504 regulations [23]. To help standardize the testing of implantable middle-ear hearing devices (IMEHDs), ASMT F2504 provides developmental regulations, and testing methods for a “standard practice” of quantifying the outputs of IMEHDs. These include criteria for cadaveric experimental methods, LDV measurement methods, etc.

**Figure 8:**
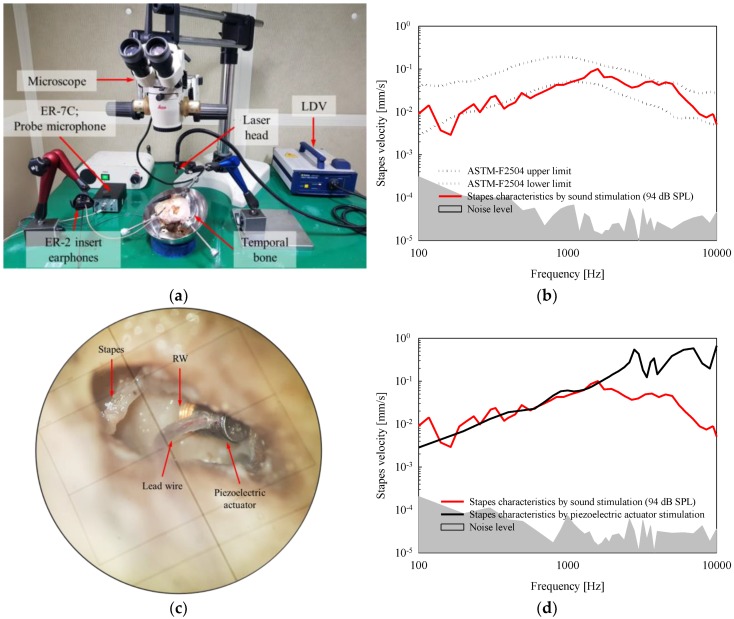
(**a**) Setup of measuring equipment for cadaveric experiment; (**b**) stapes vibrational characteristics upon sound stimulation (sound pressure level of 94 dB); (**c**) piezoelectric actuator implanted in the RW niche; (**d**) stapes vibrational characteristics upon RW stimulation using the piezoelectric actuator (6 V).

The data acquisition system was identical to the device used in the bench test. The system’s experimental environment for measuring the response characteristics of the stapes by stimulation of the TM and RW is shown in Figure 8a. To confirm the characteristics of the temporal bone, a sound pressure level (SPL) corresponding to 94 dB was applied to the TM using the ER-2 insert earphone, and the vibrational characteristics of the stapes measured. The vibrational characteristics of the stapes (red solid line) upon sound stimulation are shown in Figure 8b, and they are compared with ASTM-F2504 standards (black dotted line). The characteristics of the temporal bones used in the experiment satisfied the ASTM-F2504 standard [23]. Dental resin cement was then placed on the side of the piezoelectric housing, so as to fix the actuator once it was positioned in the RW niche (Figure 8c). After driving the actuator at 6 V, the vibrational characteristics of the stapes were measured using the LDV. Figure 8d shows the vibrational characteristics of the stapes upon RW stimulation using the piezoelectric actuator (driving voltage of 6 V). The output magnitudes of the piezoelectric actuator were equivalent to SPLs of 94 dB, 110 dB, and 115 dB for frequency ranges of below 1.6 kHz, from 1.6 kHz to 3 kHz, and above 3 kHz, respectively.

## 4. Discussion and Conclusions

In this study, a new piezoelectric actuator for an implantable middle-ear hearing aid was proposed and implemented, and its frequency characteristics were determined. The piezoelectric actuator was designed through finite element analysis so as to have an optimal frequency response for use in a middle-ear hearing aid. Once designed, a cadaveric experiment was conducted to confirm the actuator’s performance. Experimental results on a temporal bone showed that the output magnitudes of the proposed piezoelectric actuator exhibited the equivalent to SPLs of 94 dB, 110 dB, and 115 dB for frequency ranges of below 1.6 kHz, from 1.6 kHz to 3 kHz, and above 3 kHz, respectively. In particular, an equivalent to an SPL of approximately 110 dB was generated due to mechanical resonance in the region of 1 kHz to 3 kHz, where high output is required to treat sensorineural hearing loss.

**Figure 9:**
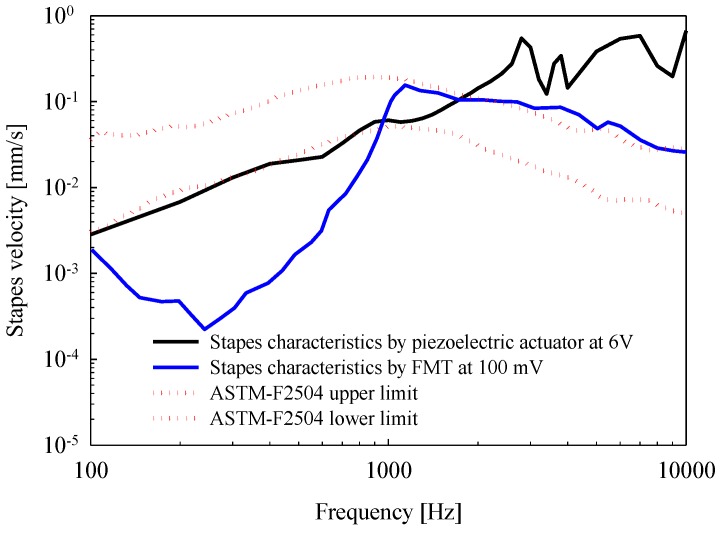
Comparison of vibrational response upon RW stimulation between the piezoelectric actuator (black solid line) and the floating mass transducer (FMT) (blue solid line, [13]).

To investigate the quality of actuator performance, we performed a comparative analysis with the results of a cadaveric experiment using an FMT reported by Nakajima et al. (2010) [13]. Figure 9 shows the vibrational characteristics of the stapes upon RW stimulation using the piezoelectric actuator (black solid line) and the FMT (blue solid line). The output characteristics of the FMT were similar to normal stapes velocities (red dotted line) in the frequency range of 1 kHz to 10 kHz (−20 dB/decade slope), and the slope of the stapes output frequency rolled off at −80 dB/decade below 1 kHz. As shown in the previous study, the low-frequency output of the FMT approach to RW-drive hearing aids was poor. In contrast, the output characteristics of the piezoelectric actuator were similar to normal stapes velocities below 1 kHz. The low-frequency output of the actuator is important, in that middle-ear hearing aids can be applied to the treatment of mixed hearing loss, and not just sensorineural hearing loss. In addition, the high output due to mechanical resonance between 1.6 kHz and 4 kHz satisfied the requirements of a middle-ear hearing aid.

The performance of the proposed piezoelectric actuator was evaluated through comparison with previous studies, and it was confirmed that the low-frequency output characteristics of the actuator were excellent, and as such, it is suitable for use as an actuator in a middle-ear hearing aid. To summarize, the proposed piezoelectric actuator offers better performance than current approaches, in terms of frequency characteristics.
